# Establishment and validation of a ferroptosis-related signature predicting prognosis and immunotherapy effect in colon cancer

**DOI:** 10.3389/fonc.2023.1201616

**Published:** 2023-05-23

**Authors:** Zhufeng Li, Fang Yuan, Xin Liu, Jianming Wei, Tong Liu, Weidong Li, Chuan Li

**Affiliations:** ^1^ Department of General Surgery, Tianjin Medical University General Hospital, Tianjin, China; ^2^ Department of Anesthesiology, Tianjin Medical University General Hospital, Tianjin, China

**Keywords:** colon cancer, ferroptosis-related gene, signature, TIME, immunotherapy

## Abstract

**Background:**

Ferroptosis, a novel form of regulating cell death, is related to various cancers. However, the role of ferroptosis-related genes (FRGs) on the occurrence and development of colon cancer (CC) needs to be further elucidated.

**Method:**

CC transcriptomic and clinical data were downloaded from TCGA and GEO databases. The FRGs were obtained from the FerrDb database. The consensus clustering was performed to identify the best clusters. Then, the entire cohort was randomly divided into the training and testing cohorts. Univariate Cox, LASSO regression and multivariate Cox analyses were used to construct a novel risk model in training cohort. The testing and the merged cohorts were performed to validate the model. Moreover, CIBERSORT algorithm analyze TIME between high- and low- risk groups. The immunotherapy effect was evaluated by analyzing the TIDE score and IPS between high- and low- risk groups. Lastly, RT-qPCR were performed to analyze the expression of the three prognostic genes, and the 2-years OS and DFS between the high- and low- risk groups of 43 clinical CC samples to further validate the value of the risk model.

**Results:**

SLC2A3, CDKN2A, and FABP4 were identified to construct a prognostic signature. Kaplan–Meier survival curves showed that OS between the high- and low-risk groups were statistically significant (p_merged_<0.001, p_training_<0.001, p_testing_<0.001). TIDE score and IPS were higher in the high-risk group (p_TIDE_<0.005, p_Dysfunction_<0.005, p_Exclusion_<0.001, p_mAb-CTLA-4 _= 3e-08, p_mAb-PD-1_ = 4.1e-10). The clinical samples were divided into high- and low- risk groups according to the risk score. There was a statistical difference in DFS (p=0.0108).

**Conclusion:**

This study established a novel prognostic signature and provided more insight into the immunotherapy effect of CC.

## Introduction

According to the 2020 Global Cancer Observatory data, colon cancer (CC) is the fourth most common cancer worldwide and the fifth leading cause of cancer-related death worldwide (1). In 2020, there were about 1.15 million new cases of CC and 580,000 deaths due to CC worldwide (1). Because there are no obvious clinical symptoms in the early stages of CC, many patients do not undergo early detection, resulting in a diagnosis of CC at an advanced stage. According to literature statistics, the 5-year relative survival rate of CC varies from 90% for stage I patients to slightly more than 10% for stage IV patients, and late diagnosis is one of the reasons for the poor prognosis of CC (2). Therefore, understanding the molecular mechanisms leading to the development of CC and identifying CC prognostic indicators are crucial for predicting the development of CC and personalizing treatment.

Ferroptosis, first proposed by Dixon in 2012, is a regulatory cell death (RCD) and depends on iron metabolism and lipid peroxidation (3). Although ferroptosis plays an important role in maintaining the survival of normal cells and tissues, it is increasingly recognized that some carcinogenic processes are related to ferroptosis, making cancer cells susceptible to ferroptosis. Ferroptosis, which is regulated by several genes, is gradually being recognized as an adaptive property to eliminate cancer cells. For example, a study has shown that SLC7A11 can reduce the proliferation of CC cells by inducing ferroptosis (4). The tumor suppressor gene p53 inhibits ferroptosis induced by erastin in colon cancer cells by blocking the activity of dipeptidyl peptidase 4, thereby inhibiting tumor development (5). Moreover, activated CD8^+^ T cells can enhance lipid peroxidation to induce ferroptosis and contribute to the antitumor effect of immunotherapy (6). Therefore, ferroptosis-related gene (FRG) has an important impact on the occurrence and development of CC and subsequent immunotherapy.

The immune components in the tumor microenvironment (TME), referred to as the tumor immune microenvironment (TIME), are closely related to tumor development, recurrence, and metastasis (7). TIME has played an important role in immunotherapy and even became a prognostic indicator. In recent decades, immunotherapy has gradually emerged as a promising area of cancer treatment, in which cytotoxic T lymphocyte antigen-4 (CTLA-4) and programmed cell death protein-1 (PD-1) is the most effective T cell immune checkpoint molecule and plays a negative immunoregulatory role (8). In patients with unresectable metastases CC, the use of PD-1 or PD-L1 to block the binding of PD-1 to ligands between tumor cells and T cells can improve immunity and is an option for advanced palliative treatment. However, immune checkpoint blockade (ICB) therapy has only a complete response rate and can show a durable response in a few CC patients, which is difficult to meet the clinical requirements of the majority of patients (9). Therefore, CC immunotherapy still needs to be explored.

Tumor markers commonly used in monitoring prognosis CC include CEA, Ras, P53, Bcl-2 and so on. The mutation or overexpression of these genes is of great importance to the prognosis of patients and the choice of treatment. However, because tumor occurrence, development and metastasis are extremely complicated, it is still inaccurate to use only the above factors for prognosis assessment. Therefore, we need to explore other prognostic markers of CC. This study clearly aimed to establish and validate the prognostic signature of CC. The signature provides an effective and practical method for clinicians to predict the survival rate of patients with CC and could predict the immunotherapy effect.

## Materials and methods

### Dataset collection

A total of 471 ferroptosis-related genes (FRGs) were downloaded from the FerrDb database (http://www.zhounan.org/ferrdb). Original counts of RNA-seq transcriptome data and clinical data derived from TCGA-COAD tissues (n=473) were obtained from The Cancer Genome Atlas (TCGA) database (https://tcga-data.nci.nih.gov/tcga/). The GSE17536 (n=177), GSE17537 (n=55), and GSE39084 (n=70) from the Gene Expression Omnibus (GEO) database (https://www.ncbi.nlm.nih.gov/geo/) were downloaded. The 8 rectal cancer samples in GSE39804 were deleted.

### Consensus clustering

The downloaded TCGA and GEO data were merged while maintaining common genes. The “sva” package of the R software was used to remove batch effects between different datasets. The expression matrix of 390 FRGs was obtained by the intersection of the expression matrix of merged cohort and the 471 FRGs. We employed consensus clustering using k-means algorithms to identify clusters. The number and stability of clusters were established by implementing the “ConsensuClusterPlus” package (10). Moreover, the categorization was repeated 1000 times to ensure its accuracy and stability (10). Lastly, the “survival” package in R software was used to evaluate the overall survival (OS) among different clusters through the Kaplan-Meier curve. Principal component analysis (PCA) could determine whether the clusters can be intuitively separated.

### Enrichment analyses

Differentially expressed genes (DEGs) were identified between different clusters using the “limma” package with an adjusted P value of 0.05 and an absolute value of |log_2_FC|=0.585. Using the Gene Ontology (GO) database, information of DEGs in cell components (CC), biological processes (BP), and molecular functions (MF) was identified (11). Using the Kyoto Encyclopedia of Genes and Genomes (KEGG) database, we were able to obtain information on possible signaling pathways, genes, and diseases associated with DEGs (12). DEGs were enriched and visualized by GO and KEGG analyzes using the packages “clusterProfiler”, “org.Hs.eg.db”, “enrichplot”, and “ggplot2”. Gene Set Variation Analysis (GSVA) enrichment analysis was used to analyze the differences in signaling pathways between different clusters (13). The”GSVA”package was used to identify the 20 most enriched signaling pathways that are differentially expressed in different clusters.

### Construction and validation of the prognostic gene signature

Univariate Cox analysis was used to identify 16 DEGs. Patients in the merged cohort were randomly divided into a training cohort and a testing cohort. Then, the Least Absolute Shrinkage and Selection Operator (LASSO) regression analysis was used to eliminate genes that might overfit the model and minimize variables (14). Multivariate Cox analysis was used to identify the prognostic genes. The risk model was constructed by three prognostic genes (SLC2A2 CDKN2A and FABP4). Risk score = Coef_gene1_ * Exp_gene1_+Coef_gene2_ * Exp_gene2_+…+Coef_genen_ * Exp_genen_. The Coef_gene_ was the risk coefficient of each gene calculated by the LASSO Cox model, and the Exp_gene_ was each gene expression level. Patients in the training cohort were divided into high- and low-risk groups based on the median risk score. Survival analysis was performed using the “survival” and “survminer” packages to compare the OS of patients in the high-and low-risk groups. Time-dependent receiver operating characteristic (ROC) curve analysis was performed with the “timeROC” package to evaluate the predictive value of the prognostic signature. Next, PCA and t-distributed stochastic neighbor embedding (t-SNE) analysis were performed using the “Rtsne” and “ggplot2” packages to determine whether the high- and low-risk groups can be intuitively separated. The testing cohort and the merged cohort were used to validate the prognostic value of the three genes.

### Value of prognostic genes

To further validate the prognostic value of the three FRGs in CC. The Tumor-immune System Interactions Database (TISIDB) database (http://cis.hku.hk/TISIDB/) was used to analyze the differences in expression of prognostic genes at different stages of CC. The Kaplan-Meier curves of the high and low expression levels of the three prognostic genes in CC were obtained from the Gene Expression Profiling Interactive Analysis (GEPIA) database (http://gepia.cancer-pku.cn/), respectively.

### Tumor immune microenvironment

To explore the differences in immune cells between high- and low-risk groups, we analyzed the correlation between tumor infiltrating immune cells (TIICs) and prognostic genes, risk score. Cell-type Identification by Estimating Relative Subsets of RNA Transcript (CIBERSORT) was an algorithm that revealed the composition ratio of 22 TIICs in TIME and was used to analyze the correlation between the risk score and the TIICs by the “limma” and “tidyverse” packages (15). The 22 TIICs of gene expression files could be obtained from the official CIBERSORT website (http://cibersort.stanford.edu/).

### Prediction of the immunotherapy effect

In order to assess the value of risk scores for the immunotherapy effect. The tumor immune dysfunction and exclusion (TIDE) analysis (http://tide.dfci.harvard.edu) was an algorithm that calculates tumor response to immune checkpoint inhibitors. The TIDE score integrated both mechanisms of T cell dysfunction and T cell rejection in tumor immune escape (16). The higher the TIDE score, the worse the immunotherapy effect and the prognosis of patients. Immunophenoscore (IPS) is used to predict the therapeutic effect of immune checkpoint inhibitors in cancer patients and is calculated based on the expression of four major types of genes that determine immunogenicity (17). The higher the IPS value, the better the patient had received immunotherapy with CTLA-4 monoclonal antibody or PD-1 monoclonal antibody (17). Obtain IPS of CTLA-4 monoclonal antibody and PD-1 monoclonal antibody in CC patients from the cancer immunome atlas (TCIA) (https://tcia.at/home), and compare immunotherapy effect between high- and low-risk groups.

### Cell line culture

The CC cell lines (HCT116, DLD-1, and LOVO) and the normal colon epithelial cell line (NCM460) were obtained from the American Type Culture Collection. These cell lines were cultured in RPMI-1640, McCoy’s 5A or F12-K medium (Gibco) containing 10% FBS (Hyclone) and 1% penicillin–streptomycin (Gibco) and incubated at 37°C with 5% CO2.

### Clinical samples collection

In this study, a retrospective cohort study was conducted to collect postoperative CC specimens and clinical data from CC patients who underwent surgery in the Department of General Surgery of Tianjin Medical University General Hospital from January 2021 to April 2021. Inclusion criteria for clinical patients: a) primary adenocarcinoma of the colon confirmed by postoperative pathology; b) negative surgical margins, complete resection of the primary lesion; c) complete clinical and pathological data, and follow-up can be completed; Exclusion criteria: a) patients with non-primary colon cancer or patients with other primary malignancies; b) patients who underwent emergency surgery; c) patients who were lost to follow-up postoperatively; d) patients whose postoperative specimens were not collected. All patients were followed up for 2 years, and follow-up ended when the patient died. Patient survival was determined from the time of surgery until the end of follow-up. According to statistics, from January 2021 to April 2021, a total of 69 patients with CC were treated surgically, except for 18 patients who did not have postoperative specimens, 3 patients who underwent emergency surgery, 3 patients who were lost to follow-up, and 2 patients with CC complicated with rectal cancer. In this study, a total of 43 patients were collected. Of the 43 CC patients, 21 patients had tumors located in the right colon, and the remaining 22 patients had tumors located in the left colon. All of them received radical tumor resection surgery, and 27 patients completed chemotherapy with capecitabine plus oxaliplatin (XELOX) after surgery. There were 3 patients with cancer-related death and 3 patients with liver metastases after CC surgery.

### Real-time quantitative polymerase chain reaction

Total RNA was extracted using TRIzol reagent (Vazyme) following the manufacturer’s protocols. The concentration of RNA was measured using a NanoDrop-2000 spectrophotometer (Thermo Fisher Scientific, Inc.) and reverse transcribed using a FastQuant RT Super mix kit (Tiangen Biotech, Co, Ltd.). Subsequently, qPCR was performed using SYBR-Green qPCR Master Mix (Bimake Biotechnology) under the following thermal cycle conditions: initial denaturation at 95°C for 15 min, followed by 40 cycles at 95°C for 10 s, 60°C for 20 s, and 72°C for 20 s. The data were quantified using 2−ΔΔCT method. The GAPDH gene was used to normalize target gene expression. The PCR primer used were as follows:

GAPDH forward primer: 5′‐TGGCACCGTCAAGGCTGAGAA‐3′;

GAPDH reverse primer: 5′‐TGGTGAAGACGCCAGTGGACTC‐3′;

SLC2A3 forward primer: 5′‐GCATCGTTGTTGGAATTCTGGT‐3′;

SLC2A3 reverse primer: 5′‐TGTAGGATAGCAGGAAGGATGG‐3′;

CDKN2A forward primer: 5′‐GGGTTTTCGTGGTTCACATCC‐3′;

CDKN2A reverse primer: 5′‐CTAGACGCTGGCTCCTCAGTA‐3′;

FABP4 forward primer: 5′‐ACTGGGCCAGGAATTTGACG‐3′;

FABP4 reverse primer: 5′‐CTCGTGGAAGTGACGCCTT‐3′.

### Statistical analysis

In this study, all statistical analyses were performed using R software (version 4.1.2) and perl-5.34.0. The t tests, nonparametric tests, and chi-square tests were used to test for differences between variables, where appropriate. The Kaplan–Meier method was used to draw survival curves for the high- and low-risk groups in the training, testing and merged cohorts, respectively, and the log-rank test was used to determine the statistical significance of differences (18). A p value <0.05 indicated a statistically significant difference.

## Results

### Identification of clusters based on the FRGs

**Figure 1:**
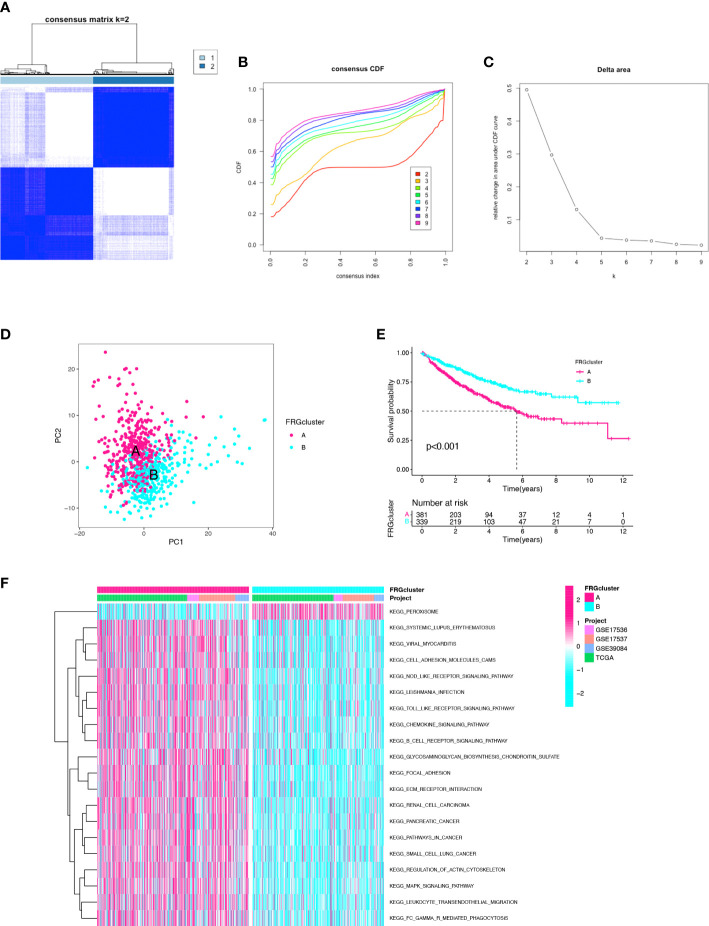
Identification of the clusters based on ferroptosis-related genes. **(A)** Merged cohort was divided into two distinct clusters using consensus clustering analysis (k = 2, repetition = 1000). **(B)** Cumulative distribution function (CDF) curve. **(C)** CDF delta area curve. **(D)** PCA of two clusters. **(E)** Comparison of K–M survival curves among the two clusters. **(F)** GSVA enrichment analysis between two clusters.

When integrating the TCGA and GEO datasets, a total of 24 repeated samples from the 13 CC patients in the TCGA cohort were deleted. The consensus clustering analysis was used to identify clusters from 743 CC patients of the merged cohort to further evaluate the prognostic genes in CC. When increasing the cluster variable (k) from 2 to 10, the intragroup correlations were found to be the highest when k=2 (**Figures 1A–C**). There were 398 patients in cluster-A and 345 patients in cluster-B. The two clusters can be clearly distinguished by PCA analysis (**Figure 1D**). The Kaplan-Meier curve was drawn between the two clusters, and the difference was statistically significant (p<0.001) (**Figure 1E**). The clinicopathological characteristics, such as age, sex, grade, pathological stage, T stage, N stage, and M stage, were compared in the heatmap between the 2 clusters, and no significant difference was found in the clinicopathological characteristics (**Figure S1**). In addition, GSVA enrichment analysis revealed that cluster-B was enriched in the peroxisome pathway compared with cluster-A (**Figure 1F**). These results indicated that the two clusters identified based on FRGs accurately reflected the prognostic difference.

### Functional enrichment analysis of DEGs

**Figure 2:**
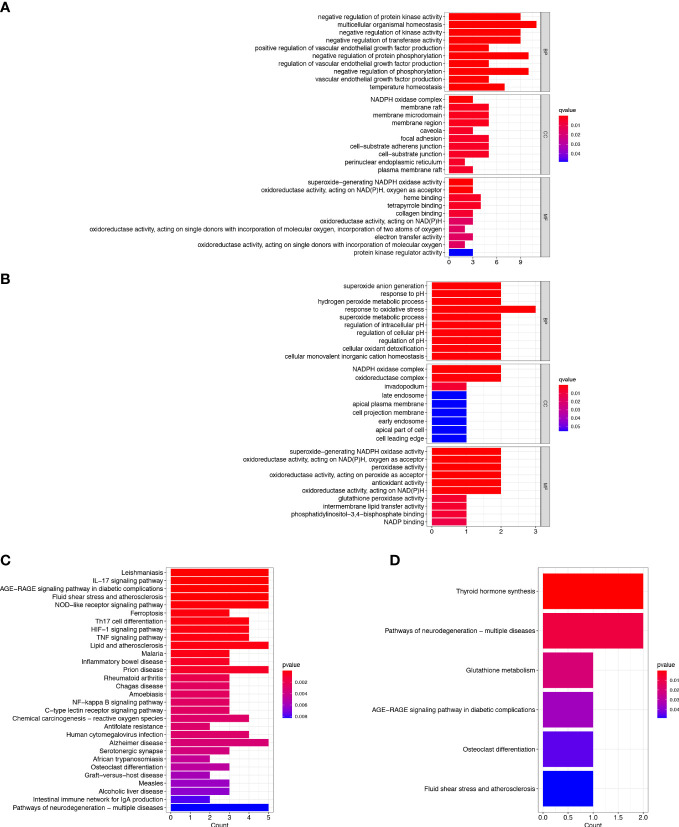
Enrichment Analyses. **(A)** GO analysis of risk genes in DEGs. **(B)** GO analysis of protective genes in DEGs. **(C)** KEGG analysis of risk genes in DEGs. **(D)** KEGG analysis of protective genes in DEGs.

To explore the potential biological behavior of ferroptosis in the two clusters, DEGs between cluster-A and cluster-B were analyzed. There were 35 DEGs found between the two clusters, including 4 protective genes and 31 risk genes. GO and KEGG enrichment analyses were implemented to gain insights into signal pathways of clusters. The GO enrichment analysis indicated that risk genes were predominantly enriched in multicellular organismal homeostasis (biological process), membrane raft (cellular component) and heme binding (molecular function) (**Figure 2A**). The GO enrichment analysis indicated that protective genes were predominantly enriched in response to oxidative stress (biological process), NADPH oxidase complex (cellular component) and superoxide−generating NADPH oxidase activity (molecular function) (**Figure 2B**). In addition, the KEGG pathway analysis showed that the risk genes were enriched in the ferroptosis, IL-17 signaling pathway, NOD-like receptor signaling pathway and HIF-1 signaling pathway (**Figure 2C**). The KEGG pathway analysis showed that the protective genes were enriched in the thyroid hormone synthesis (**Figure 2D**). The DEGs were mainly enriched in ferroptosis-related pathways, and ferroptosis might play a dual effect on tumor cells.

### Construction of prognostic three-gene signature

**Figure 3:**
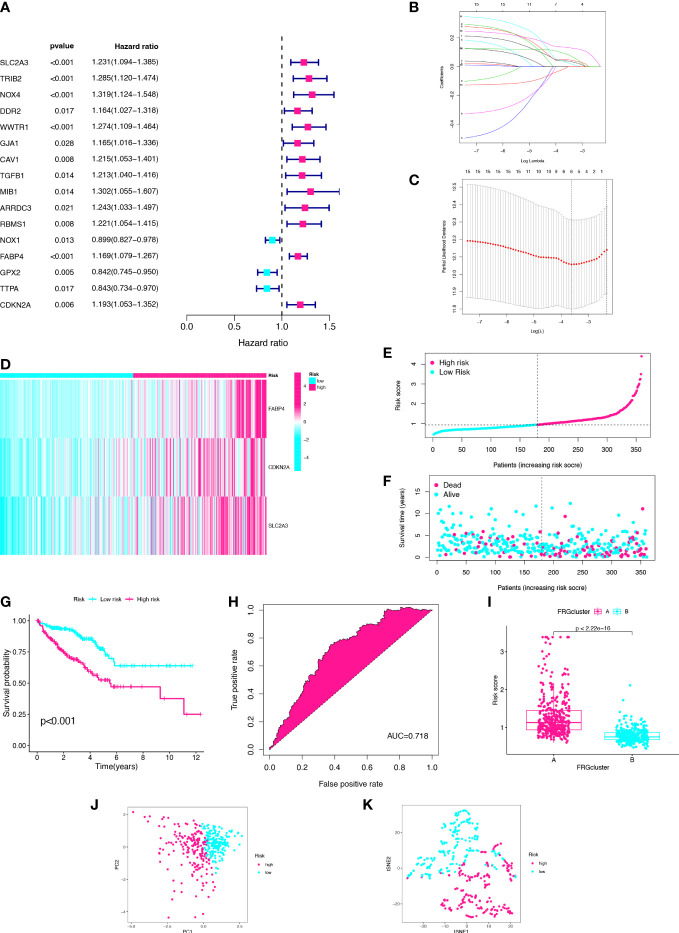
Establishment of three-gene prognostic signature using the training cohort. **(A)** Univariate Cox analysis of DEGs. **(B)** LASSO regression analysis of the 16 DEGs. **(C)** Cross-validation method to select optimal genes. **(D)** Heatmap of three gene expression levels. **(E)** Distribution and median value of risk scores. **(F)** Distribution of risk scores and alive status. **(G)** K–M curve. **(H)** ROC curve. **(I)** The two clusters have significant differences in risk score. **(J)** PCA analysis. **(K)** t-SNE analysis.

We constructed a risk signature to better diagnose the prognosis of CC. With filtering the 23 CC samples with missing survival status, missing survival time and 0 survival time in the TCGA database. A total of 720 CC samples were used for the construction and validation of the risk model. Using the univariate Cox analysis, 16 DEGs were identified as prognostic signatures **(** **Figure 3A**). Patients in the merged cohort were randomly divided into training cohort (n=360) and testing cohort (n=360). Based on the expression levels of the 16 DEGs and clinical data, three genes significantly associated with the prognosis of CC were identified by applying the LASSO regression analysis and the multivariate Cox analysis, including SLC2A3, CDKN2A, and FABP4 (**Figures 3B, C**). The risk score was calculated according to the following formula: Risk score = SLC2A3 Exp*0.136407939746576 + CDKN2A Exp *0.175273043748396 + FABP4 Exp *0.165558961729006. According to the risk model, the heatmap, risk score distribution plot, and survival status scatter plot were used to evaluate the model (**Figures 3D–F**; **Table S1**). The CC patients in the training cohort were divided into high- and low-risk groups based on the median risk score. The number of patients with death status increased with the risk score. The risk score of cluster-A was significantly higher than the risk score of cluster-B (p<2.22e-16, **Figure 3G**). Then, the Kaplan-Meier plot showed that low-risk patients had a significantly better OS than high-risk patients (p<0.001) (**Figure 3H**). In this study, the area under curve (AUC) of training cohort predicting the sensitivity and specificity of 5-year OS was 0.718 (**Figure 3I**). Finally, after dimensionality reduction by the PCA and the t-SNE analyses, it was found that the CC samples could clearly visualize the distribution between the high- and low-risk groups (**Figures 3J, K**). We identified three FRGs and constructed a prognostic signature that could well predict the prognosis of CC patients.

### Validation of the prognostic signature in the merged cohort and testing cohort

**Figure 4:**
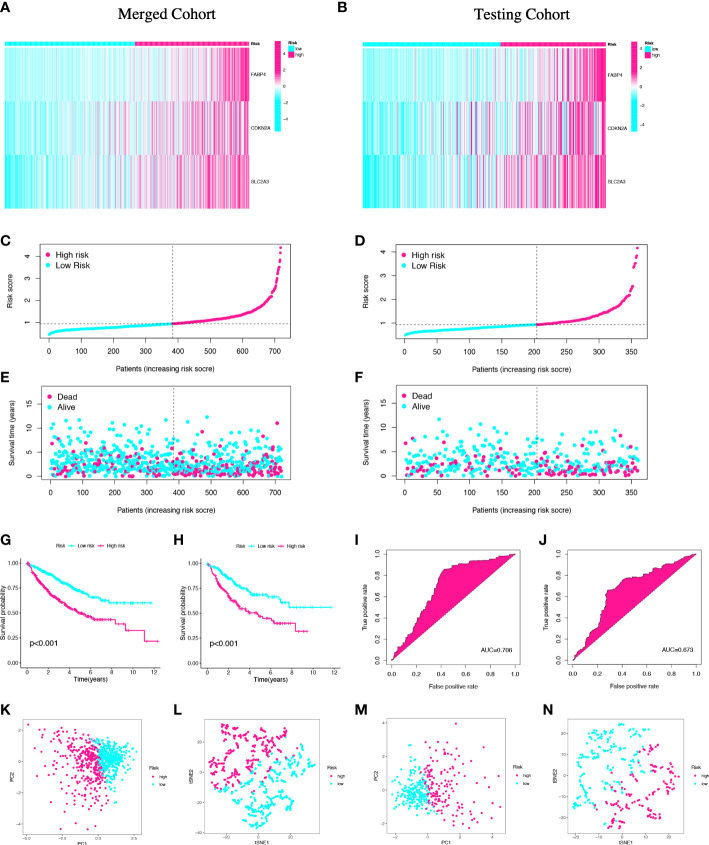
Validation of the prognostic signature in the merged and testing cohorts. Risk heatmap **(A, B)**, risk grouping **(C, D)**, survival status **(E, F)**, Kaplan–Meier curves **(G, H)**, ROC curves **(I, J)**, PCA and t-SNE analyses **(K-N)** of different risk groups in the merged cohort and the testing cohort.

In order to validate the prognostic value of the risk model, the same methods were performed in the merged cohort and testing cohort (**Figures 4A–F**; **Table S1**). Then, the Kaplan–Meier plot revealed that the high-risk group had a significantly worse OS compared with the low-risk group in the both groups (p_merged_<0.001, p_testing_<0.001) (**Figures 4G, H**). The AUCs for 5-year OS were 0.706 and 0.673, respectively, indicating that the risk model had a strong prognostic value for CC patients in the merged cohort and testing cohort (**Figures 4I, J**). Lastly, the high- and low-risk groups can be clearly distinguished by PCA and t-SNE analyses (**Figures 4K–N**). Both the merged cohort and the testing cohort had well validated the value of the prognostic signature.

### The value of three ferroptosis-related genes in colon cancer

**Figure 5:**
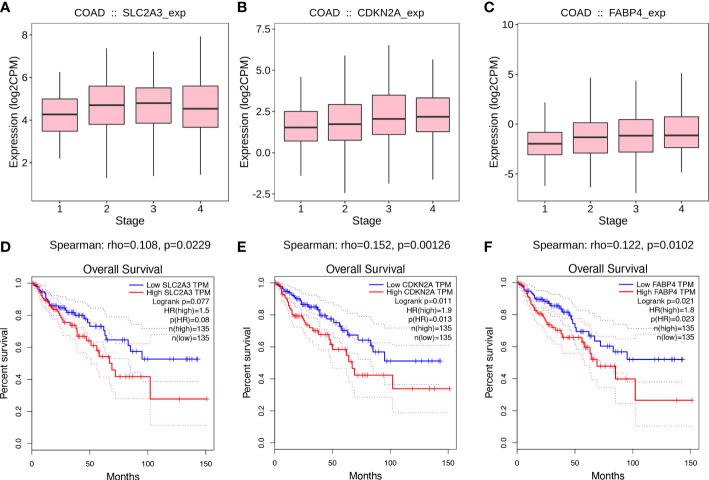
Value of prognostic genes. **(A-C)** Associations between prognostic genes expression and stage across colon cancer from the TISIDB database. **(D-F)** Kaplan-Meier survival curves from the GEPIA database.

To identify the potential value of SLC2A3, CDKN2A, and FABP4 on the prognosis of CC, we generated gene expression differential diagrams of CC stages I-IV and Kaplan-Meier survival curves from the TISIDB database and the GEPIA database, respectively. The expression levels of SLC2A3, CDKN2A and FABP4 genes increased with the growth of CC stage (rho_SLC2A3 =_ 0.108, p_SLC2A3 =_ 0.0229, rho_CDKN2A_=0.152, p_CDKN2A_=0.00126, rho_FABP4 =_ 0.122, p_FABP4 =_ 0.0102) (**Figures 5A–C**).High expression of CDKN2A and FABP4 were associated with poor prognosis in CC, but there was no statistical difference in survival curves of SLC2A3 (p_SLC2A3 =_ 0.077, p_CDKN2A_=0.011, p_FABP4 =_ 0.021) (**Figures 5D–F**). The analysis results of the TISIDB and the GEPIA databases further proved that the three FRGs were all risk genes in CC.

### Tumor immune microenvironment in colon cancer

**Figure 6:**
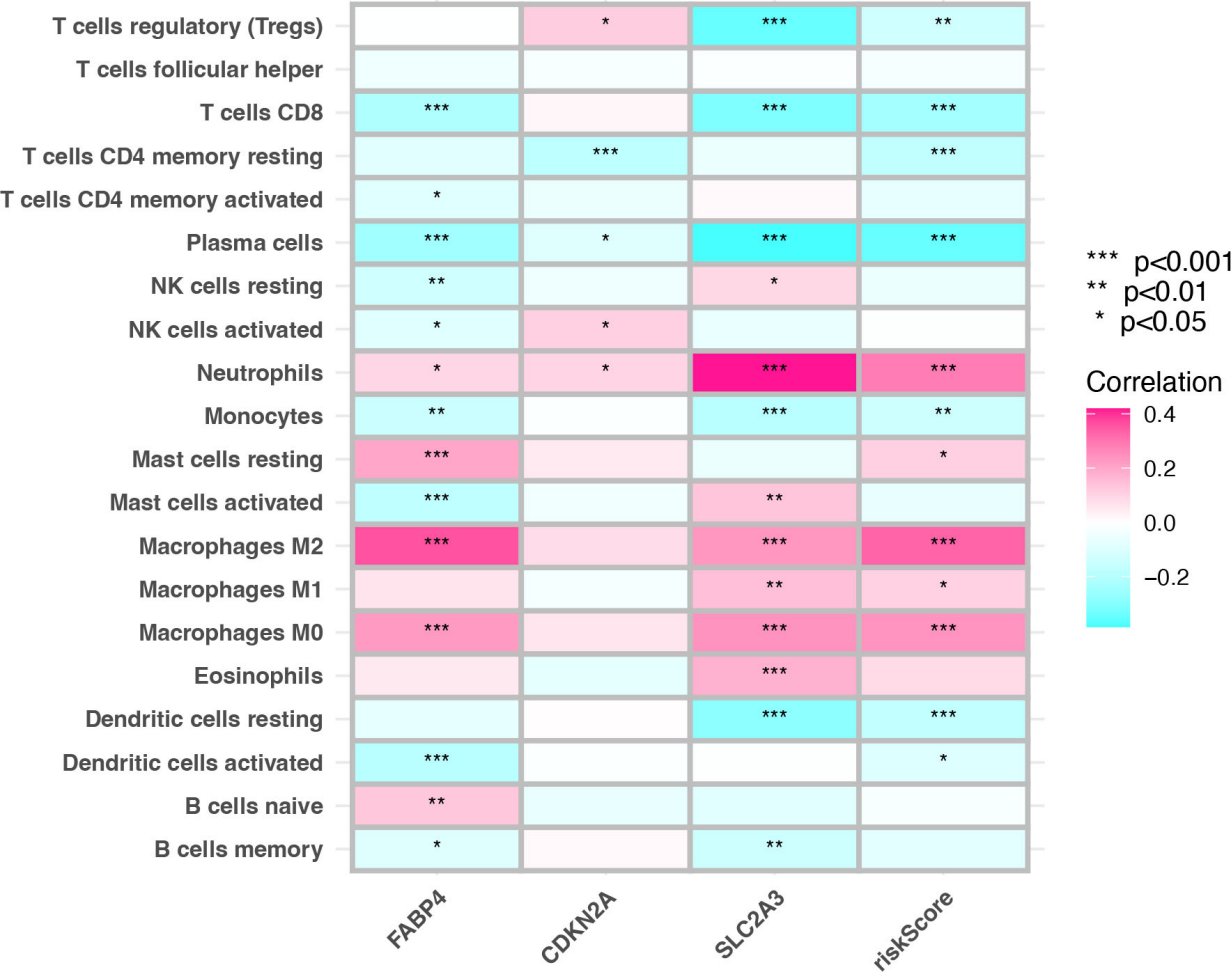
Correlation between tumor infiltrating immune cells and prognostic genes, risk score.

The status of the tumor TIME determines the immunotherapy effect. The results suggest that there is a close correlation between prognostic genes and TIICs was investigated. The correlation between prognostic genes and TIICs was essentially consistent with the risk score. The risk score was mainly positively correlated with neutrophils and macrophages infiltration and negatively correlated with infiltration of CD4^+^T cells, CD8^+^T cells, regulatory T cells, B cells, plasma cells and dendritic cells (P <0.001) (**Figure 6**). In this study, there are significant correlations between SLC2A3, CDKN2A, FABP4, risk score, and TIICs.

### Risk model predicts the immunotherapy effect in colon cancer

**Figure 7:**
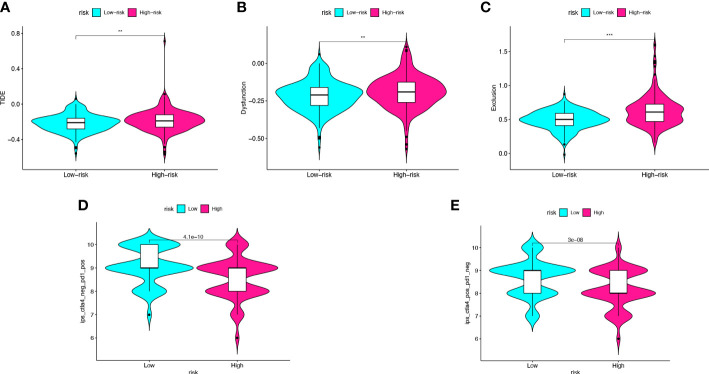
Risk score predicts the immunotherapy effect of colon cancer. **(A–C)** The TIDE score, dysfunction score, and exclusion score of the high- and low-risk score groups. **(D, E)** Prediction of the immunotherapy effect of high- and lowrisk groups to the anti-CTLA4 or anti-PD1 based on IPS. **p < 0.005, ***p < 0.001.

To further validate the value of the risk model for immunotherapy, we predicted response to immunotherapy in high- and low-risk groups. TIDE analysis calculated response to immunotherapy from CC in the TCGA cohort. The TIDE score, dysfunction score, and exclusion score of the high-risk group were significantly higher than those of the low-risk group (p_TIDE_<0.005, p_Dysfunction_<0.005, p_Exclusion_<0.001) (**Figures 7A–C**). The IPS score was used to analyze potential response to immunotherapy. The results showed that the low-risk group had higher IPS score after treatment with CTLA-4 monoclonal antibody or PD-1 monoclonal antibody (p_mAb-CTLA-4 =_ 3e-08, p_mAb-PD-1 =_ 4.1e-10) (**Figures 7D, E**). Therefore, we predicted that the immunotherapy effect might be better in low-risk groups than in high-risk groups.

### Validation of the risk model in colon cancer clinical samples

**Figure 8:**
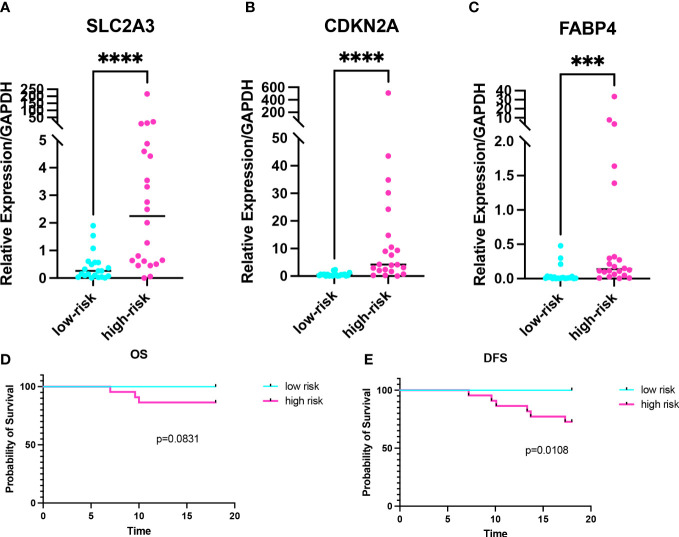
Validation of the risk model in colon cancer clinical samples. **(A-C)** Expression levels of prognostic genes in the high- and low-risk groups. **(D, E)** Kaplan-Meier curve of 2-years OS and 2-years DFS in the high- and low-risk groups. ***p < 0.001, ****p < 0.0001.

In order to further validate the prognostic value of the risk model, we performed RT-qPCR on 43 CC samples and obtained the relative expression level of prognostic genes in each sample (**Table S2**). The risk score of 43 CC samples was calculated. There were no statistically significant differences in clinicopathological characteristics between high- and low-risk groups (**Table S3**). The expression levels of prognostic genes were higher in the high-risk group than in the low-risk group (p_SLC2A3 =_ 0.0002, p_CDKN2A_<0.0001, p_FABP4 =_ 0.0001) (**Figures 8A–C**). Combined with the 2-years follow-up data of 43 CC patients, the OS and DFS were calculated between the high- and low-risk groups. There was no statistical difference in OS, but there was a statistical difference in DFS (p_OS_=0.0831, p_DFS_=0.0108) (**Figures 8D, E**). Validation of the risk model with clinical samples further demonstrated the prognostic value of the risk model for CC patients, and the high-risk group patients might have the worse OS and DFS.

### Expression analysis of prognostic genes in GEPIA database and cell lines

**Figure 9:**
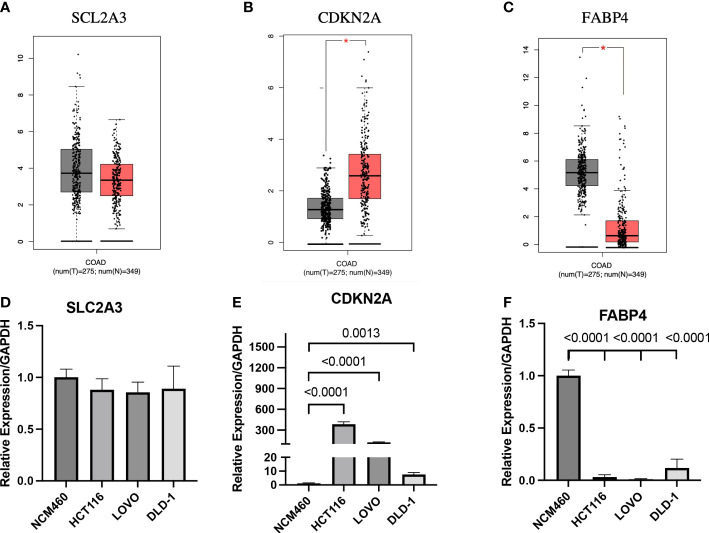
Expression analysis of prognostic genes in GEPIA database and cell lines **(A-C)** Expression analysis of the three genes in GEPIA database. **(D-F)** Expression analysis of the three genes between normal cell line and colon cancer cell lines. *p < 0.01.

In this study, the expression differences between normal tissues and cancer tissues were also compared. The diagram for difference analysis of expression of three prognostic genes in normal and cancer tissues was downloaded from the GEPIA database. The result showed that there was no significant difference in the expression of SLC2A3 between normal and cancer tissues; the expression of CDKN2A in cancer tissues was higher than that in normal tissues (p<0.01); the expression of FABP4 in cancer tissues was lower than that in normal tissues (p<0.01) (**Figures 9A–C**). The expression of three prognostic genes was verified by RT-qPCR in CC cell lines. The expression results of the three prognostic genes between CC cell lines (HCT116, DLD-1, and LOVO) and normal cell line (NCM460) were consistent with the expression differences between tissues in the GEPIA database (p_CDKN2A-HCT116_<0.0001, p_CDKN2A- LOVO_<0.0001, p_CDKN2A-DLD-1 =_ 0.0013, p_FABP4-HCT116_<0.0001, p_FABP4-LOVO_<0.0001, p_FABP4-DLD-1_<0.0001) (**Figures 9D–F**). Therefore, CDKN2A and FABP4 not only had the prognostic value for CC patients, but also were expected to become the therapeutic targets of CC in the future.

## Discussion

Colon cancer (CC) is one of the leading causes of cancer-related death worldwide, and its increasing morbidity and mortality have led to CC patients with poor prognosis (1). Both the TNM stage of the tumor and the clinicopathological characteristics of the patient are closely related to the prognosis of CC (19). However, the diagnosis is often made late, because the initial symptoms of CC are not obvious. In many patients, the diagnosis is made at an advanced stage. In recent years, with the development of molecular biology, biomarkers have become an important part of clinical diagnosis and treatment. Valuable biomarkers can predict the prognosis of patients and open a new direction for exploring the study of disease development (20).

Ferroptosis is a form of RCD characterized by iron overload, accumulation of lipid reactive oxygen species (ROS), and inactivation of the cellular antioxidant glutathione (GSH) (21). A growing number of evidence shows that ferroptosis is closely related to the cancer occurrence, development and inhibition (21–23). N-acetyltransferase 10 promotes the progression of CC, by inhibiting ferroptosis through N4-acetylation and stabilization of ferroptosis suppressor protein 1 (FSP1) mRNA (24). *In vitro* studies confirmed that combined treatment with β-elements and cetuximab induced ferroptosis in KRAS mutant CC cells HCT116 and LOVO (25). Thus, it was found that ferroptosis may not only play a role as an anti-tumor method, but also that absence of ferroptosis may promote tumorigenesis. However, the understanding of FRGs in the pathogenesis of CC is still insufficient. Therefore, we analyze FRGs to construct a novel risk model to predict the prognosis of patients with CC.

Solute Carrier Family 2 Member 3 (SLC2A3), also known as Glucose Transporter 3 (GLUT3), is a FRG involved in glucose transmembrane transport. Glucose transporter (GLUT) protein expression is often increased in cancer cells, and upregulation of the SLC2A encoding GLUT protein is associated with poor prognosis in many cancers (26–28). A study pointed out that upregulation of SLC2A3 was associated with decreased OS and DFS in colorectal cancer patients (29), which indicated that SLC2A3 played an important role in the prognosis of CC patients. Highly expressed SLC2A3 can increase the expression of PD-L1 in CC, and the prognosis of CC patients with overexpression PD-L1 is worse (26).

Cyclin Dependent Kinase Inhibitor 2A (Cyclin Dependent Kinase Inhibitor 2A, CDKN2A) is an important cell cycle regulator that plays a regulatory role in cell proliferation and apoptosis, and mainly encodes two proteins, p16INK4A and p14ARF (30). A study has found that the overexpression of CDKN2A is an independent prognostic factor for colorectal cancer, and its overexpression can induce the occurrence of epithelial-mesenchymal transition, thereby promoting the further development of tumors (31).CDKN2A also can increase the sensitivity of cells to ROS-triggered ferroptosis and promote carcinogenesis by enhancing p53-dependent transactivation and ferroptosis (32).

Fatty Acid Binding Protein 4 (FABP4) is a class of intracellular lipid transporter mainly expressed in adipocytes and macrophages (33).FABP4 can participate in lipid transfer between adipocytes and tumor cells, induce fatty acid oxidation pathway to promote tumor growth (34).Chen et al. also pointed out that overexpression FABP4 is a risk factor for colorectal cancer and can be used as a potential biomarker to diagnose colorectal cancer (35). In other similar studies, FABP4 also affects the prognosis of CC as a risk gene (36, 37).

Although the mechanism of tumor susceptibility to ferroptosis has been a research focus in the past few years, the relationship and regulation between TIME and ferroptosis is still elusive. The TIME, regulated by TIICs, has a critical effect on the occurrence and development of CC. For example, neutrophils can promote tumor growth by promoting tumor angiogenesis or suppressing anti-tumor immunity (38). Uribe-Querol et al. believe that the ROS produced by neutrophils in the early stages of tumor development is not sufficient to kill tumor cells but promotes tumor proliferation through genotoxicity and DNA damage (39). Both types of M1 and M2 macrophages are polarized from M0 macrophages but play opposite roles in tumor development (40). The M2 macrophage can promote the motility, migration and invasion of CC cells through its exosomes (41). M1 macrophages secrete large amounts of proinflammatory factors and tumor necrosis factors to play the biological functions of promoting inflammation and inhibiting cancer (40). Yang et al. indicate that iron accumulation in ferroptosis is correlated with abundant iron stores in M1 macrophages, and high levels of ROS generated by ferroptosis may also promote polarization of macrophages toward M1 (42). This study suggests a high correlation between ferroptosis and M1 macrophages and also explains the positive correlation of risk score with M1 macrophages. In a disease state such as cancer, regulatory cells (Tregs) become an impediment as they compromise the anti-tumor response of the host by dampening the efficiency of T-effector cells (43). However, Tregs suppress bacterial-driven inflammation that promotes carcinogenesis, which benefits the host (44). Therefore, Tregs may be a double-edged sword in tumor development. B cells play a crucial role in humoral immunity by producing antibodies, while they also enhance T cell-mediated immunity by acting as APCs (45). CD8^+^T cells, also known as cytotoxic T lymphocytes (CTLs), mainly mediate cytotoxic activity by inducing tumor cell apoptosis, which is considered to be the most critical component of antitumor immunity (46). Interestingly, we found that CDKN2A appeared to be less correlated with TIICs than the other two genes and the risk score. A study has found that up-regulated CDKN2A affects intercellular signal transduction through the TGF-β pathway to induce immunosuppression (47). Luo et al. inferred the high expression of CDKN2A affects the immune microenvironment, downregulates immune activity, and thus promotes the recurrence of metastatic CC (48). In summary, the risk score is inversely correlated with the infiltration of CD8^+^T cells, B cells, CD4^+^T cells, but positively correlated with the infiltration of neutrophils and macrophages. Due to the complexity and multifunctionality of TIICs, we need to further investigate the relationship between the three prognostic genes and TIICs in the future.

In recent decades, immunotherapy has gradually emerged a promising field of cancer treatment, among which T lymphocyte-based tumor immunotherapy becoming an effective tool for cancer treatment. Activated CD8^+^T cells can enhance lipid peroxidation in tumor cells, thereby inducing ferroptosis and contributing to the antitumor efficacy of immunotherapy (6). Although PD -1 or CTLA-4 monoclonal antibody may show some efficacy in colorectal cancer with DNA mismatch repair deficiency, more accurate biomarkers to predict immunotherapy effect remain to be found. Therefore, we hypothesized that FRGs have an important impact on the immunotherapy of CC. It is particularly important to explore the relationship between FRG and CC immunotherapy and predict the immunotherapy effect in patients with CC. The TIDE score corresponds to T cell dysfunction and exclusion, which has been proven to be significant in predicting the efficacy of immune checkpoint blockade (16). IPS is a reliable method for predicting anti-CTLA-4 and anti-PD-1 immunotherapy (17). In this study, both the IPS score and the TIDE score in predicting the immunotherapy effect showed that the immunotherapy effect was worse in high-risk groups than in low-risk groups. This risk model also has predictive value for the immunotherapy effect in CC patients.

In order to further validate the accuracy of the risk model, a total of 43 clinical samples from CC patients from January 2021 to April 2021 were selected for RT-qPCR to detect the expression of prognostic genes and calculate the risk score. The expression of SLC2A3, CDKN2A, and FABP4 was higher in the high-risk group of clinical samples than in the low-risk group, and there was statistical significance. Combined with the clinicopathological data of the patients, the prognosis of the patients in the high-risk group and the low-risk group was poor. There was no statistical difference in OS, but there was a statistical difference in DFS. This might be due to the small number of patients and the short postoperative follow-up period. In the future, the sample size needs to be increased and the follow-up time of patients needs to be extended before the results can be verified. Thus, the risk model composed of SLC2A3, CDKN2A, and FABP4 has some value for the clinical evaluation of the prognosis of CC.

To further explore the value of prognostic genes, we analyzed the differences in expression levels of prognostic genes between normal tissues (or normal cell line) and CC tissues (or CC cell lines). CDKN2A was significantly overexpressed in CC tissues compared with normal colon tissues, whereas the expression of FABP4 was significantly lower in CC tissues compared with normal colon tissues. There was no difference in the expression of SLC2A3 between CC tissues and normal colon tissues. The above results are similar to the expression differences of prognosis genes between CC cell lines (HCT116, DLD-1, and LOVO) and normal colon cell line (NCM460) in our RT-qPCR results. Most importantly, the analysis results of some studies on the expression of prognostic genes are also consistent with the results of the GEPIA database or our RT-qPCR (35, 49, 50). Because the screening of DEGs in this study was carried out between two CC tissues with different prognosis, there are uncertainties in expression levels of prognostic genes between normal tissues (or normal cell line) and CC tissues (or CC cell lines). In future studies still need to study the mechanism of occurrence and development of prognostic genes in CC for further explanation. In summary, we believe that CDKN2A and FABP4 not only have a certain value in the prognosis of CC patients, but also are expected to become targets for future CC treatment options.

Compared with other studies, we constructed the risk model by consensus clustering based on FRGs. In addition, a total of 471 FRGs were included in this study, which is more extensive than other ferroptosis-related signatures (51, 52). And we predicted the effect of CC immunotherapy based on the prognostic signature. Finally, the value of the risk model was validated using external databases and clinical samples. To our knowledge, this study discovered and established a new prognostic signature of CC, which playes a role in evaluating the prognosis and predicting the immunotherapy effect in CC patients.

This study has some limitations. First, with the continuous progress of ferroptosis research, more and more FRGs may occur in the future, and the different FRGs included in the risk model may lead to different risk model results. Second, this risk model is only used to predict the prognosis of CC patients and the immunotherapy effect, but not all low-risk patients have a good prognosis or respond positively to immunotherapy. Finally, the number of clinical samples used to verify the risk model is small and the follow-up time is not long, so it is necessary to further increase the sample size and extend the follow-up time for further verification.

## Conclusion

SLC2A3, CDKN2A, and FABP4 are risk genes in CC, and they are involved in the three key steps of ferroptosis in energy metabolism (SLC2A3), iron metabolism (CDKN2A) and lipid metabolism (FABP4). The risk model, which was constructed using the ferroptosis-related genes (SLC2A3, CDKN2A, and FABP4), has good predictive value for the prognosis of CC patients. This risk model can also predict the effect of CC immunotherapy. CC patients with high risk scores had higher TIDE scores, more T-cell dysfunction and rejection, lower IPS and poorer response to immunotherapy with CTLA-4 mAb or PD-1 mAb.

## Data availability statement

The original contributions presented in the study are included in the article/**Supplementary Material**. Further inquiries can be directed to the corresponding author.

## Ethics statement

Ethical review and approval was not required for the study on human participants in accordance with the local legislation and institutional requirements. Written informed consent for participation was not required for this study in accordance with the national legislation and the institutional requirements.

## Author contributions

ZL, FY, and XL contributed to the conception and design of the study. JW contributed to the writing and editing the manuscript. CL, WL, and TL provided administrative, technical, or material support. All authors contributed to the article and approved the submitted version.

## Supplementary material

Supplementary Figure 1 Heatmap of clinical features and ferroptosis-related genes expressions between clusters.

## References

[1] Sung H Ferlay J Siegel RL Laversanne M Soerjomataram I Jemal A. Global cancer statistics 2020: GLOBOCAN estimates of incidence and mortality worldwide for 36 cancers in 185 countries. CA Cancer J Clin (2021) 71(3):209–49. doi: 10.3322/caac.21660 33538338

[2] Dekker E Tanis PJ Vleugels JLA Kasi PM Wallace MB. Colorectal cancer. Lancet (2019) 394(10207):1467–80. doi: 10.1016/s0140-6736(19)32319-0 31631858

[3] Dixon SJ Lemberg KM Lamprecht MR Skouta R Zaitsev EM Gleason CE. Ferroptosis: an iron-dependent form of nonapoptotic cell death. Cell (2012) 149(5):1060–72. doi: 10.1016/j.cell.2012.03.042 PMC3367386 22632970

[4] Xu X Zhang X Wei C Zheng D Lu X Yang Y. Targeting SLC7A11 specifically suppresses the progression of colorectal cancer stem cells *via* inducing ferroptosis. Eur J Pharm Sci (2020) 152:105450. doi: 10.1016/j.ejps.2020.105450 32621966

[5] Xie Y Zhu S Song X Sun X Fan Y Liu J. The tumor suppressor p53 limits ferroptosis by blocking DPP4 activity. Cell Rep (2017) 20(7):1692–704. doi: 10.1016/j.celrep.2017.07.055 28813679

[6] Wang W Green M Choi JE Gijon M Kennedy PD Johnson JK. CD8(+) T cells regulate tumour ferroptosis during cancer immunotherapy. Nature (2019) 569(7755):270–4. doi: 10.1038/s41586-019-1170-y PMC6533917 31043744

[7] Fu T Dai LJ Wu SY Xiao Y Ma D Jiang YZ. Spatial architecture of the immune microenvironment orchestrates tumor immunity and therapeutic response. J Hematol Oncol (2021) 14(1):98. doi: 10.1186/s13045-021-01103-4 34172088 PMC8234625

[8] Yaghoubi N Soltani A Ghazvini K Hassanian SM Hashemy SI. PD-1/PD-L1 blockade as a novel treatment for colorectal cancer. BioMed Pharmacother (2019) 110:312–8. doi: 10.1016/j.biopha.2018.11.105 30522017

[9] Binnewies M Roberts EW Kersten K Chan V Fearon DF Merad M. Understanding the tumor immune microenvironment (TIME) for effective therapy. Nat Med (2018) 24(5):541–50. doi: 10.1038/s41591-018-0014-x PMC5998822 29686425

[10] Wilkerson MD Hayes DN. ConsensusClusterPlus: a class discovery tool with confidence assessments and item tracking. Bioinformatics (2010) 26(12):1572–3. doi: 10.1093/bioinformatics/btq170 PMC2881355 20427518

[11] Peng J Wang H Lu J Hui W Wang Y Shang X. Identifying term relations cross different gene ontology categories. BMC Bioinf (2017) 18(Suppl 16):573. doi: 10.1186/s12859-017-1959-3 PMC5751813 29297309

[12] Kanehisa M GS. KEGG- kyoto encyclopedia of genes and genomes. Nucleic Acids Res (2000) 28(1):27–30. doi: 10.1093/nar/28.1.27 10592173 PMC102409

[13] Hänzelmann S CR Guinney J. GSVA-gene set variation analysis for microarray and RNA-seq data. BMC Bioinf (2013) 14:7. doi: 10.1186/1471-2105-14-7 PMC3618321 23323831

[14] Xiang ZJ Wang Y Ramadge PJ. Screening tests for lasso problems. IEEE Trans Pattern Anal Mach Intell (2017) 39(5):1008–27. doi: 10.1109/TPAMI.2016.2568185 27187950

[15] Newman AM Liu CL Green MR Gentles AJ Feng W Xu Y. Robust enumeration of cell subsets from tissue expression profiles. Nat Methods (2015) 12(5):453–7. doi: 10.1038/nmeth.3337 PMC4739640 25822800

[16] Jiang P Gu S Pan D Fu J Sahu A Hu X. Signatures of T cell dysfunction and exclusion predict cancer immunotherapy response. Nat Med (2018) 24(10):1550–8. doi: 10.1038/s41591-018-0136-1 PMC6487502 30127393

[17] Charoentong P Finotello F Angelova M Mayer C Efremova M Rieder D. Pan-cancer immunogenomic analyses reveal genotype-immunophenotype relationships and predictors of response to checkpoint blockade. Cell Rep (2017) 18(1):248–62. doi: 10.1016/j.celrep.2016.12.019 28052254

[18] Koletsi D Pandis N. Survival analysis, part 2: Kaplan-Meier method and the log-rank test. Am J Orthod Dentofacial Orthop (2017) 152(4):569–71. doi: 10.1016/j.ajodo.2017.07.008 28962743

[19] Karim S Brennan K Nanji S Berry SR Booth CM. Association between prognosis and tumor laterality in early-stage colon cancer. JAMA Oncol (2017) 3(10):1386–92. doi: 10.1001/jamaoncol.2017.1016 PMC5710504 28594974

[20] Califf RM. Biomarker definitions and their applications. Exp Biol Med (Maywood) (2018) 243(3):213–21. doi: 10.1177/1535370217750088 PMC5813875 29405771

[21] Wang Y Wei Z Pan K Li J Chen Q. The function and mechanism of ferroptosis in cancer. Apoptosis (2020) 25(11-12):786–98. doi: 10.1007/s10495-020-01638-w 32944829

[22] Sui X Zhang R Liu S Duan T Zhai L Zhang M. RSL3 drives ferroptosis through GPX4 inactivation and ROS production in colorectal cancer. Front Pharmacol (2018) 9:1371. doi: 10.3389/fphar.2018.01371 30524291 PMC6262051

[23] Jiang X Stockwell BR Conrad M. Ferroptosis: mechanisms, biology and role in disease. Nat Rev Mol Cell Biol (2021) 22(4):266–82. doi: 10.1038/s41580-020-00324-8 PMC8142022 33495651

[24] Zheng X Wang Q Zhou Y Zhang D Geng Y Hu W. N-acetyltransferase 10 promotes colon cancer progression by inhibiting ferroptosis through N4-acetylation and stabilization of ferroptosis suppressor protein 1 (FSP1) mRNA. Cancer Commun (Lond) (2022) 42(12):1347–66. doi: 10.1002/cac2.12363 PMC9759759 36209353

[25] Chen P Li X Zhang R Liu S Xiang Y Zhang M. Combinative treatment of beta-elemene and cetuximab is sensitive to KRAS mutant colorectal cancer cells by inducing ferroptosis and inhibiting epithelial-mesenchymal transformation. Theranostics (2020) 10(11):5107–19. doi: 10.7150/thno.44705 PMC7163451 32308771

[26] Ha TK Chi SG. CAV1/caveolin 1 enhances aerobic glycolysis in colon cancer cells *via* activation of SLC2A3/GLUT3 transcription. Autophagy (2012) 8(11):1684–5. doi: 10.4161/auto.21487 PMC3494600 22874559

[27] Younes M BR Stephenson M Gondo M Cagle PT. Overexpression of Glut1 and Glut3 in stage I nonsmall cell lung carcinoma is associated with poor survival. Cancer (1997) 80(6):1046–51. doi: 10.1002/(sici)1097-0142(19970915)80:6<1046::aid-cncr6>3.0.co;2-7 9305704

[28] Chai YJ Yi JW Oh SW Kim YA Yi KH Kim JH. Upregulation of SLC2 (GLUT) family genes is related to poor survival outcomes in papillary thyroid carcinoma: analysis of data from the cancer genome atlas. Surgery (2017) 161(1):188–94. doi: 10.1016/j.surg.2016.04.050 27842912

[29] Kim E Jung S Park WS Lee JH Shin R Heo SC. Upregulation of SLC2A3 gene and prognosis in colorectal carcinoma: analysis of TCGA data. BMC Cancer (2019) 19(1):302. doi: 10.1186/s12885-019-5475-x 30943948 PMC6446261

[30] Padhi SS Roy S Kar M Saha A Roy S Adhya A. Role of CDKN2A/p16 expression in the prognostication of oral squamous cell carcinoma. Oral Oncol (2017) 73:27–35. doi: 10.1016/j.oraloncology.2017.07.030 28939073

[31] Shi WK Li YH Bai XS Lin GL. The cell cycle-associated protein CDKN2A may promotes colorectal cancer cell metastasis by inducing epithelial-mesenchymal transition. Front Oncol (2022) 12:834235. doi: 10.3389/fonc.2022.834235 35311137 PMC8929760

[32] Chen D Tavana O Chu B Erber L Chen Y Baer R. NRF2 is a major target of ARF in p53-independent tumor suppression. Mol Cell (2017) 68(1):224–32 e4. doi: 10.1016/j.molcel.2017.09.009 28985506 PMC5683418

[33] Furuhashi M Hotamisligil GS. Fatty acid-binding proteins: role in metabolic diseases and potential as drug targets. Nat Rev Drug Discov (2008) 7(6):489–503. doi: 10.1038/nrd2589 18511927 PMC2821027

[34] Nieman KM Romero IL Van Houten B Lengyel E. Adipose tissue and adipocytes support tumorigenesis and metastasis. Biochim Biophys Acta (2013) 1831(10):1533–41. doi: 10.1016/j.bbalip.2013.02.010 PMC3742583 23500888

[35] Zhang Y Zhao X Deng L Li X Wang G Li Y. High expression of FABP4 and FABP6 in patients with colorectal cancer. World J Surg Oncol (2019) 17(1):171. doi: 10.1186/s12957-019-1714-5 31651326 PMC6814121

[36] Cai JW Huang XM Li XL Qin S Rong YM Chen X. An 11-gene signature for the prediction of systemic recurrences in colon adenocarcinoma. Gastroenterol Rep (Oxf) (2021) 9(5):451–60. doi: 10.1093/gastro/goab023 PMC8560041 34733531

[37] Liu J Lan Y Tian G Yang J. A systematic framework for identifying prognostic genes in the tumor microenvironment of colon cancer. Front Oncol (2022) 12:899156. doi: 10.3389/fonc.2022.899156 35664768 PMC9161737

[38] Li MO Wolf N Raulet DH Akkari L Pittet MJ Rodriguez PC. Innate immune cells in the tumor microenvironment. Cancer Cell (2021) 39(6):725–9. doi: 10.1016/j.ccell.2021.05.016 34129817

[39] Uribe-Querol E Rosales C. Neutrophils in cancer: two sides of the same coin. J Immunol Res (2015) 2015:983698. doi: 10.1155/2015/983698 26819959 PMC4706937

[40] Mantovani A Sica A Locati M. Macrophage polarization comes of age. Immunity (2005) 23(4):344–6. doi: 10.1016/j.immuni.2005.10.001 16226499

[41] Lan J Sun L Xu F Liu L Hu F Song D. M2 macrophage-derived exosomes promote cell migration and invasion in colon cancer. Cancer Res (2019) 79(1):146–58. doi: 10.1158/0008-5472.CAN-18-0014 30401711

[42] Yang Y Wang Y Guo L Gao W Tang TL Yan M. Interaction between macrophages and ferroptosis. Cell Death Dis (2022) 13(4):355. doi: 10.1038/s41419-022-04775-z 35429990 PMC9013379

[43] Verma A Mathur R Farooque A Kaul V Gupta S Dwarakanath BS. T-Regulatory cells in tumor progression and therapy. Cancer Manag Res (2019) 11:10731–47. doi: 10.2147/CMAR.S228887 PMC6935360 31920383

[44] Whiteside TL. What are regulatory T cells (Treg) regulating in cancer and why? Semin Cancer Biol (2012) 22(4):327–34. doi: 10.1016/j.semcancer.2012.03.004 PMC3385925 22465232

[45] Farhood B Najafi M Mortezaee K. CD8(+) cytotoxic T lymphocytes in cancer immunotherapy: a review. J Cell Physiol (2019) 234(6):8509–21. doi: 10.1002/jcp.27782 30520029

[46] Zhang Y Zhang Z. The history and advances in cancer immunotherapy: understanding the characteristics of tumor-infiltrating immune cells and their therapeutic implications. Cell Mol Immunol (2020) 17(8):807–21. doi: 10.1038/s41423-020-0488-6 PMC7395159 32612154

[47] Massague J. TGFβ in cancer. Cell (2008) 134(2):215–30. doi: 10.1016/j.cell.2008.07.001 PMC3512574 18662538

[48] Luo Z Chen X Zhang Y Huang Z Zhao H Zhao J. Development of a metastasis-related immune prognostic model of metastatic colorectal cancer and its usefulness to immunotherapy. Front Cell Dev Biol (2020) 8:577125. doi: 10.3389/fcell.2020.577125 33585439 PMC7876250

[49] Noguchi Y OT Marat D Yoshikawa T Saitoh A Doi C Fukuzawa K. Expression of facilitative glucose transporter 1 mRNA in colon cancer was not regulated by k-ras. Cancer Lett (2000) 154(2):137–42. doi: 10.1016/s0304-3835(00)00354-2 10806301

[50] Lee M Sup Han W Kyoung Kim O Hee Sung S Sun Cho M Lee SN. Prognostic value of p16INK4a and p14ARF gene hypermethylation in human colon cancer. Pathol Res Pract (2006) 202(6):415–24. doi: 10.1016/j.prp.2005.11.011 16675157

[51] Nie J Shan D Li S Zhang S Zi X Xing F. A novel ferroptosis related gene signature for prognosis prediction in patients with colon cancer. Front Oncol (2021) 11:654076. doi: 10.3389/fonc.2021.654076 34046350 PMC8144717

[52] Zhu J Kong W Xie Z. Expression and prognostic characteristics of ferroptosis-related genes in colon cancer. Int J Mol Sci (2021) 22(11). doi: 10.3390/ijms22115652 PMC8199073 34073365

