# Supplementary figures and images for: Establishment and validation of a ferroptosis-related signature predicting prognosis and immunotherapy effect in colon cancer
Source: Front Oncol. 2023 May 23;13:1201616. doi: 10.3389/fonc.2023.1201616 (PMC10243598; doi:10.3389/fonc.2023.1201616)

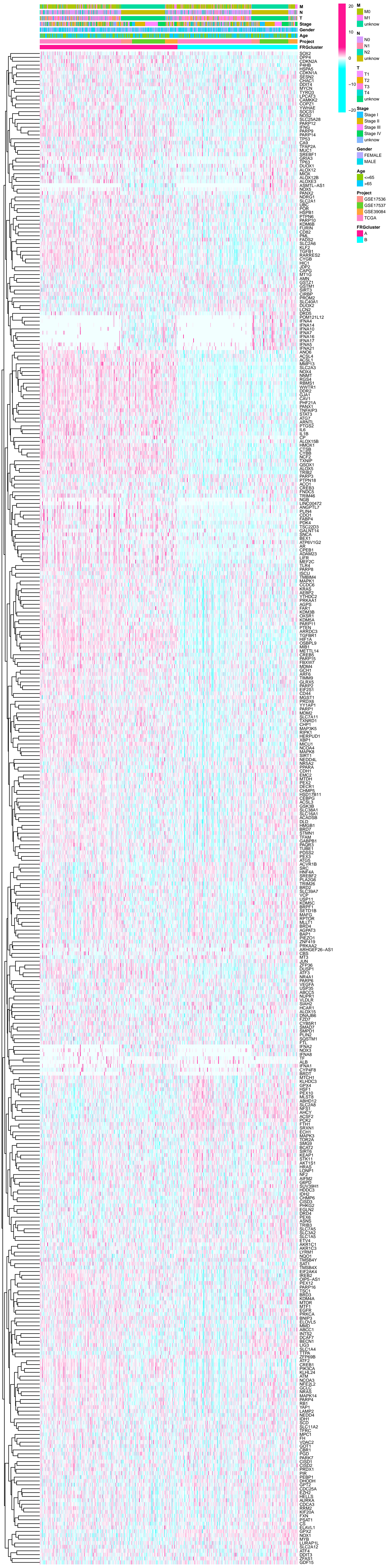

Supplement: Supplementary Figure 1 — Heatmap of clinical features and ferroptosis-related genes expressions between clusters. [file DataSheet_1.pdf]
