# Supplementary material for: Establishment and validation of a ferroptosis-related signature predicting prognosis and immunotherapy effect in colon cancer
Source: Front Oncol. 2023 May 23;13:1201616. doi: 10.3389/fonc.2023.1201616 (PMC10243598; doi:10.3389/fonc.2023.1201616)
Supplement: Supplementary file 2 [file Table_1.docx]

Table 1. Distribution of risk scores and alive status in colon cancer patients

| Colon cancer patients | Low risk group(%) | High risk group(%) | χ^2^ | p |
| --- | --- | --- | --- | --- |
| Training cohort |  |  | 17.13 | <0.0001 |
| Alive | 152 | 118 |  |  |
| Dead | 28 | 62 |  |  |
| Testing cohort |  |  | 16.39 | <0.0001 |
| Alive | 155 | 87 |  |  |
| Dead | 49 | 69 |  |  |
| Merged cohort |  |  | 31.28 | <0.0001 |
| Alive | 307 | 205 |  |  |
| Dead | 77 | 131 |  |  |
