# Supplementary material for: Establishment and validation of a ferroptosis-related signature predicting prognosis and immunotherapy effect in colon cancer
Source: Front Oncol. 2023 May 23;13:1201616. doi: 10.3389/fonc.2023.1201616 (PMC10243598; doi:10.3389/fonc.2023.1201616)
Supplement: Supplementary file 3 [file Table_2.docx]

Table 2. Relative gene expression and risk score of clinical colon cancer patients

| Sample | SLC2A3 | CDKN2A | FABP4 | Risk score | Group | Status | OS  (month) | DFS  (month) |
| --- | --- | --- | --- | --- | --- | --- | --- | --- |
| No.7 | 0.0082 | 0.0976 | 0.0049 | 0.0190 | low risk | 0 | 18 | 18 |
| No.10 | 0.0284 | 0.0990 | 0.0004 | 0.0213 | low risk | 0 | 18 | 18 |
| No.38 | 0.0705 | 0.1885 | 0.0004 | 0.0427 | low risk | 0 | 18 | 18 |
| No.34 | 0.1002 | 0.1927 | 0.0041 | 0.0481 | low risk | 0 | 18 | 18 |
| No.6 | 0.0071 | 0.0691 | 0.2986 | 0.0625 | low risk | 0 | 18 | 18 |
| No.30 | 0.2602 | 0.1661 | 0.0109 | 0.0664 | low risk | 0 | 18 | 18 |
| No.37 | 0.0919 | 0.4994 | 0.0118 | 0.1020 | low risk | 0 | 18 | 18 |
| No.19 | 0.6105 | 0.1016 | 0.0222 | 0.1048 | low risk | 0 | 18 | 18 |
| No.20 | 0.0326 | 0.6547 | 0.0016 | 0.1195 | low risk | 0 | 18 | 18 |
| No.27 | 0.0366 | 0.6571 | 0.0203 | 0.1235 | low risk | 0 | 18 | 18 |
| No.11 | 0.1445 | 0.6106 | 0.0003 | 0.1268 | low risk | 0 | 18 | 18 |
| No.29 | 0.3202 | 0.5177 | 0.0103 | 0.1361 | low risk | 0 | 18 | 18 |
| No.12 | 0.3688 | 0.6626 | 0.0061 | 0.1675 | low risk | 0 | 18 | 18 |
| No.22 | 0.4940 | 0.5643 | 0.0352 | 0.1721 | low risk | 0 | 18 | 18 |
| No.2 | 1.0776 | 0.2288 | 0.0280 | 0.1917 | low risk | 0 | 18 | 18 |
| No.43 | 0.5687 | 0.5068 | 0.2113 | 0.2014 | low risk | 0 | 18 | 18 |
| No.24 | 1.5403 | 0.0522 | 0.0063 | 0.2203 | low risk | 0 | 18 | 18 |
| No.35 | 0.1528 | 1.2931 | 0.0066 | 0.2486 | low risk | 0 | 18 | 18 |
| No.13 | 1.8977 | 0.3586 | 0.4793 | 0.4011 | low risk | 0 | 18 | 18 |
| No.31 | 0.2586 | 2.2998 | 0.0310 | 0.4436 | low risk | 0 | 18 | 18 |
| No.21 | 0.5607 | 2.0959 | 0.0137 | 0.4462 | low risk | 0 | 18 | 18 |
| No.15 | 0.8001 | 2.0038 | 0.0469 | 0.4682 | high risk | 0 | 18 | 18 |
| No.17 | 2.7559 | 0.6940 | 0.1137 | 0.5164 | high risk | 0 | 18 | 18 |
| No.42 | 0.6141 | 2.9352 | 0.0083 | 0.5997 | high risk | 1 | 10.1 | 10.1 |
| No.14 | 0.4543 | 3.1099 | 0.0032 | 0.6077 | high risk | 0 | 18 | 13.3 |
| No.39 | 2.4932 | 0.2348 | 1.3884 | 0.6112 | high risk | 0 | 18 | 18 |
| No.32 | 0.6372 | 4.1184 | 0.0975 | 0.8250 | high risk | 0 | 18 | 18 |
| No.41 | 0.4541 | 4.2413 | 0.1485 | 0.8300 | high risk | 0 | 18 | 18 |
| No.9 | 4.4214 | 0.2544 | 1.6390 | 0.9191 | high risk | 1 | 7.2 | 7.2 |
| No.5 | 4.8740 | 1.7077 | 0.2127 | 0.9994 | high risk | 0 | 18 | 18 |
| No.23 | 4.5926 | 2.3965 | 0.3193 | 1.0994 | high risk | 0 | 18 | 18 |
| No.40 | 0.5034 | 7.6464 | 0.0618 | 1.4193 | high risk | 0 | 18 | 18 |
| No.26 | 5.4978 | 3.9673 | 0.1091 | 1.4634 | high risk | 0 | 18 | 18 |
| No.16 | 1.2758 | 9.3064 | 0.1748 | 1.8344 | high risk | 1 | 9.6 | 9.6 |
| No.25 | 2.0044 | 8.9728 | 0.1133 | 1.8651 | high risk | 0 | 18 | 18 |
| No.36 | 3.5398 | 10.4874 | 0.2728 | 2.3664 | high risk | 0 | 18 | 18 |
| No.3 | 0.0030 | 14.7493 | 0.0263 | 2.5903 | high risk | 0 | 18 | 17.3 |
| No.8 | 0.0537 | 0.0090 | 33.4304 | 5.5450 | high risk | 0 | 18 | 18 |
| No.18 | 3.3057 | 34.8308 | 0.2966 | 6.6058 | high risk | 0 | 18 | 18 |
| No.1 | 19.4270 | 24.2191 | 0.0082 | 6.8968 | high risk | 0 | 18 | 18 |
| No.33 | 0.6493 | 43.5287 | 0.1261 | 7.7400 | high risk | 0 | 18 | 18 |
| No.28 | 216.8800 | 30.1607 | 3.1700 | 35.3946 | high risk | 0 | 18 | 18 |
| No.4 | 9.5478 | 507.2039 | 7.6723 | 91.4857 | high risk | 0 | 18 | 13.7 |
