# Supplementary material for: Establishment and validation of a ferroptosis-related signature predicting prognosis and immunotherapy effect in colon cancer
Source: Front Oncol. 2023 May 23;13:1201616. doi: 10.3389/fonc.2023.1201616 (PMC10243598; doi:10.3389/fonc.2023.1201616)
Supplement: Supplementary file 4 [file Table_3.docx]

Table 3. Comparison of clinicopathological features between high- and low-risk groups in clinical patients with colon cancer

| Clinicopathological features | Low risk group(%) | High risk group(%) | χ^2^ | p |
| --- | --- | --- | --- | --- |
| Gender |  |  | Fisher | 0.2231 |
| Male | 11（61.11） | 7 （38.89） |  |  |
| Female | 10（40.00） | 15（60.00） |  |  |
| Age (Year) |  |  | Fisher | >0.9999 |
| <60 | 5 （50.00） | 5 （50.00） |  |  |
| ≥60 | 16（48.48） | 17（51.52） |  |  |
| T |  |  | 0.5593 | 0.7411 |
| T2 | 2 （40.00） | 3 （60.00） |  |  |
| T3 | 11（55.00） | 9 （45.00） |  |  |
| T4 | 8 （44.44） | 10（55.56） |  |  |
| N |  |  | 4.403 | 0.1106 |
| N0 | 19（57.58） | 14（42.42） |  |  |
| N1 | 1 （16.67） | 5 （83.33） |  |  |
| N2 | 1 （25.00） | 3 （75.00） |  |  |
| M |  |  | Fisher | 0.6069 |
| M0 | 20（51.28） | 19（48.72） |  |  |
| M1 | 1 （25.00） | 3 （75.00） |  |  |
| Stage |  |  | 6.298 | 0.0980 |
| Stage I | 2 （66.67） | 1 （33.33） |  |  |
| Stage II | 16（61.54） | 10（38.46） |  |  |
| Stage III | 2 （20.00） | 8 （80.00） |  |  |
| Stage IV | 1 （25.00） | 3 （75.00） |  |  |
| Tumor site |  |  | Fisher | 0.3660 |
| Right colon | 12（57.14） | 9 （42.86） |  |  |
| Left colon | 9 （40.91） | 13（59.09） |  |  |
| Adjuvant chemotherapy |  |  | Fisher | >0.9999 |
| Completion | 14（51.85） | 13（48.15） |  |  |
| No | 8 （50.00） | 8 （50.00） |  |  |
